# Interaction of cyclodextrins with pyrene-modified polyacrylamide in a mixed solvent of water and dimethyl sulfoxide as studied by steady-state fluorescence

**DOI:** 10.3762/bjoc.8.150

**Published:** 2012-08-16

**Authors:** Akihito Hashidzume, Yongtai Zheng, Akira Harada

**Affiliations:** 1 Department of Macromolecular Science, Graduate School of Science, Osaka University, 1-1 Machikaneyama-cho, Toyonaka, Osaka 560-0043, Japan; 2 Japan Science and Technology Agency (JST), Core Research for Evolutional Science and Technology (CREST), 7 Gobancho, Chiyoda-ku, Tokyo 102-0076, Japan

**Keywords:** cyclodextrins, interaction, pyrene-modified polyacrylamide, steady-state fluorescence, water/DMSO mixed solvent

## Abstract

The interaction of β- and γ-cyclodextrins (β-CD and γ-CD, respectively) with polyacrylamide modified with pyrenyl (Py) residues (pAAmPy) was investigated in a mixed solvent of water and dimethyl sulfoxide (DMSO) by steady-state fluorescence. In the absence of CD, the fluorescence spectra indicated that the formation of Py dimers became less favorable with increasing volume fraction of DMSO (*x*_DMSO_). The fluorescence spectra at varying *x*_DMSO_ and CD concentrations indicated that β-CD and γ-CD included monomeric and dimeric Py residues, respectively. Using the fluorescence spectra, equilibrium constants of the formation of Py dimers and the complexation of β-CD and γ-CD with Py residues were roughly estimated based on simplified equilibrium schemes.

## Introduction

Cyclodextrins (CDs) are cyclic oligomers composed of glucopyranose units linked through α-1,4-glycoside bonding. They bear a tapered structure with a narrower rim of primary hydroxy groups and a wider rim of secondary hydroxy groups. CDs of 6, 7, and 8 glucopyranose units are called α-CD, β-CD, and γ-CD, respectively. CDs have a hydrophilic exterior and a rather hydrophobic cavity, and thus, recognize guest compounds of a size and shape matching their cavity, to form inclusion complexes [1–5]. Since CDs are nontoxic, they have been utilized in a variety of fields, including food additives, cosmetics, and personal care items [6–12]. In the past decade, the formation of inclusion complexes of CDs with guest residues attached on water-soluble polymers has attracted increasing interest from a number of research groups because these systems are applicable to stimuli-responsive systems [13–18].

**Scheme 1:**
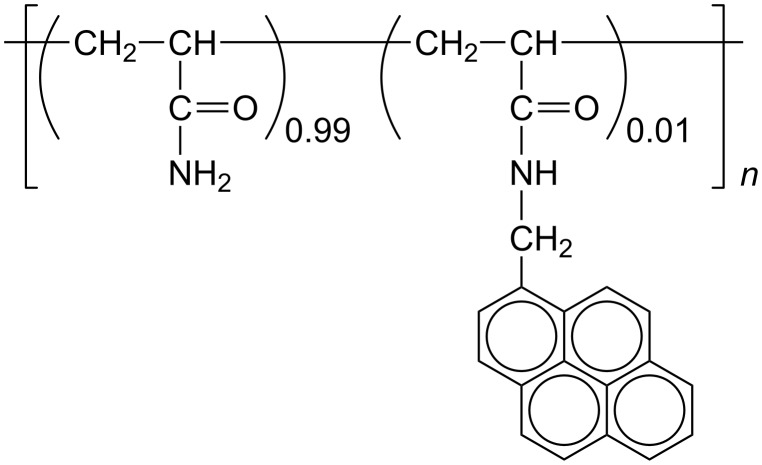
Structure of pAAmPy.

We have been working on the interaction of CDs with water-soluble polymers bearing various guest residues, including linear, branched, and cyclic aliphatics, as well as aromatics [19–21], and realized stimuli-responsive hydrogels [22–27] and macroscopic assemblies based on molecular recognition [28–31]. Aromatic residues absorb light to become excited, and subsequently they can transfer energy and electrons. The interaction of CDs with water-soluble polymers carrying aromatic residues may allow one to construct functional systems that convert photo energy based on molecular recognition. Among aromatic compounds, pyrene is the most examined as a fluorescence probe or label because it shows a relatively high fluorescence quantum yield and a relatively long fluorescence lifetime in both monomer and excimer states [32–33]. Since pyrene is very hydrophobic, it may tend to form aggregates, e.g., dimers, in aqueous solutions. It is also known that pyrene forms inclusion complexes with β-CD and γ-CD in different manners; β-CD includes monomeric pyrene whereas γ-CD includes dimeric pyrene [34–36]. Recently, we have demonstrated this selectivity switching on macroscopic molecular recognition for polyacrylamide-based gels carrying pyrenyl (Py) and CD residues, by changing the composition of a mixed solvent of water and dimethyl sulfoxide (DMSO) [37]. In the present study, the interaction of β-CD and γ-CD with Py-modified polyacrylamide (pAAmPy, Scheme 1) was investigated in the water/DMSO mixed solvent of varying composition by steady-state fluorescence to elucidate the mechanism of the selectivity switching.

## Results

**Figure 1:**
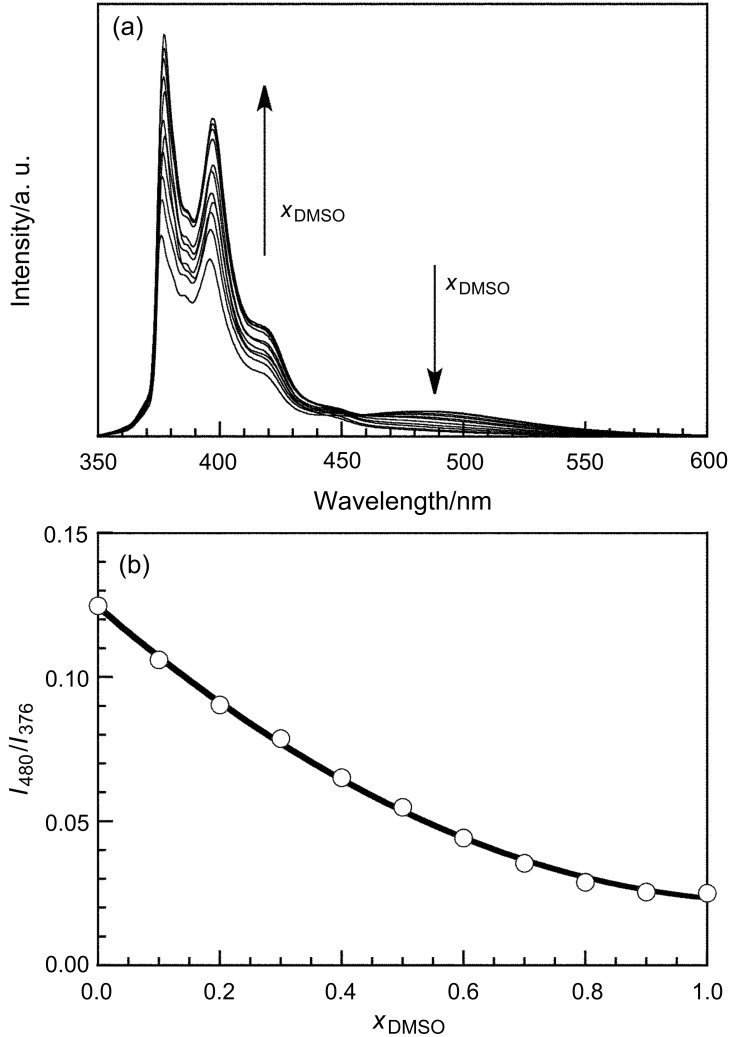
Steady-state fluorescence spectra for 0.04 g L^−1^ pAAmPy at varying *x*_DMSO_ from 0 to 1 with excitation at 335 nm (a) and *I*_480_/*I*_376_ as a function of *x*_DMSO_ (b).

Figure 1a demonstrates the steady-state fluorescence spectra measured for 0.04 g L^−1^ pAAmPy (5 μM in Py residue) at varying volume fractions of DMSO (*x*_DMSO_) in the water/DMSO mixed solvent in the absence of CD. At *x*_DMSO_ = 0 (i.e., in water), the spectrum exhibits not only emission bands ascribable to monomeric Py in the region of 370–430 nm, but also a broad band assignable to a Py excimer around 480 nm, indicating that Py residues tend to form dimers because of the hydrophobicity. It is likely that Py residues associate intramolecularly under the dilute conditions (0.04 g L^−1^) in this study. These spectra indicate that the intensity of excimer fluorescence decreases whereas that of monomer fluorescence increases with increasing *x*_DMSO_. This observation indicates that the formation of Py dimer becomes less favorable, because the Py residue becomes more solvophilic with *x*_DMSO_. Using the spectra, the ratios (*I*_480_/*I*_376_) of the intensities at 480 and 376 nm, which are predominantly due to the Py excimer and monomer, respectively, were calculated and plotted in Figure 1b against *x*_DMSO_. *I*_480_/*I*_376_ decreases monotonously from 0.125 to 0.025 with increasing *x*_DMSO_ from 0 to 1.

**Figure 2:**
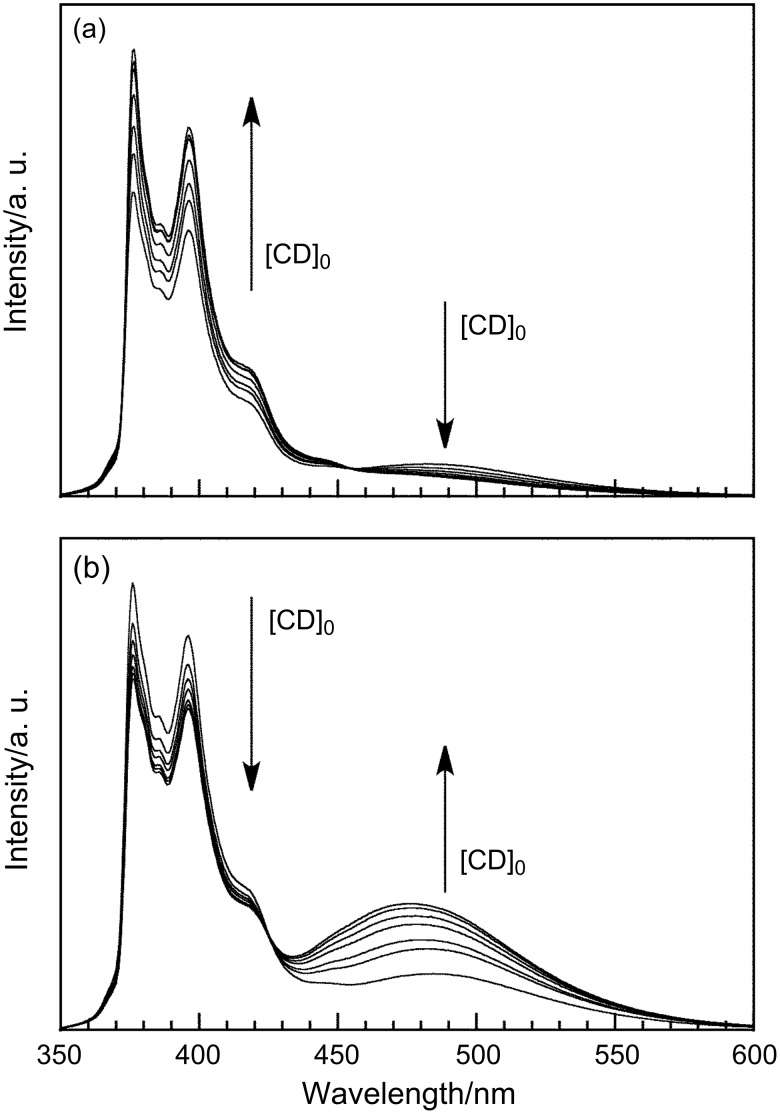
Steady-state fluorescence spectra for 0.04 g L^−1^ pAAmPy with excitation at 335 nm in the presence of varying concentrations of β-CD at *x*_DMSO_ = 0.1 (a) and of γ-CD at *x*_DMSO_ = 0 (b).

**Figure 3:**
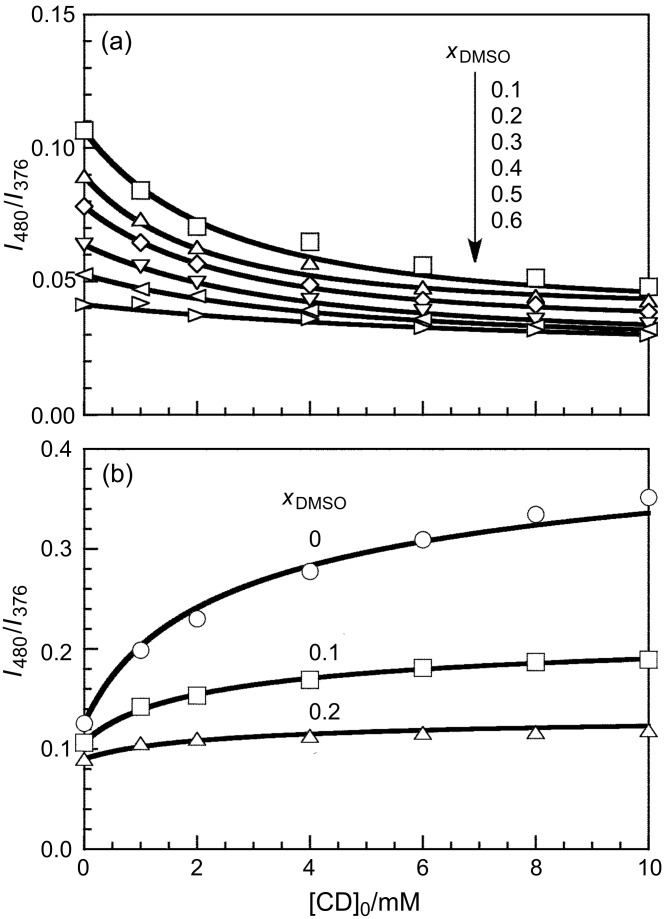
*I*_480_/*I*_376_ as a function of [CD]_0_ for β-CD/pAAmPy (a) and γ-CD/pAAmPy (b) at different *x*_DMSO_.

The interaction of β-CD and γ-CD with pAAmPy was also investigated at varying *x*_DMSO_ by steady-state fluorescence. Figure 2 exhibits fluorescence spectra for the β-CD/pAAmPy system at *x*_DMSO_ = 0.1 and for the γ-CD/pAAmPy system at *x*_DMSO_ = 0 as typical examples, showing remarkable tendencies. In the spectra of the β-CD/pAAmPy system at *x*_DMSO_ = 0.1, the intensity of Py excimer fluorescence decreases whereas that of Py monomer fluorescence increases with the increasing concentration of CD ([CD]_0_), indicating that β-CD forms inclusion complexes with monomeric Py residues, and dimeric Py residues dissociate to the monomers. In the spectra of the γ-CD/pAAmPy system at *x*_DMSO_ = 0, on the other hand, the intensity of the excimer fluorescence increases whereas that of the monomer fluorescence decreases with increasing [CD]_0_, indicating that γ-CD forms inclusion complexes with dimeric Py residues, and monomeric Py residues further associate to form the dimers. Using the steady-state fluorescence spectra, *I*_480_/*I*_376_ values were calculated. Figure 3 compares *I*_480_/*I*_376_ as a function of [CD]_0_ for the β-CD/pAAmPy system at *x*_DMSO_ = 0.1–0.6 and for the γ-CD/pAAmPy system at *x*_DMSO_ = 0–0.2. At other *x*_DMSO_, *I*_480_/*I*_376_ was practically independent of [CD]_0_, indicative of no significant interaction of β-CD or γ-CD with pAAmPy. For the β-CD/pAAmPy system (Figure 3a), *I*_480_/*I*_376_ decreases with increasing [CD]_0_ at *x*_DMSO_ = 0.1–0.6. For the γ-CD/pAAmPy system (Figure 3b), on the other hand, *I*_480_/*I*_376_ increases with [CD]_0_ at *x*_DMSO_ = 0–0.2.

## Discussion

**Scheme 2:**
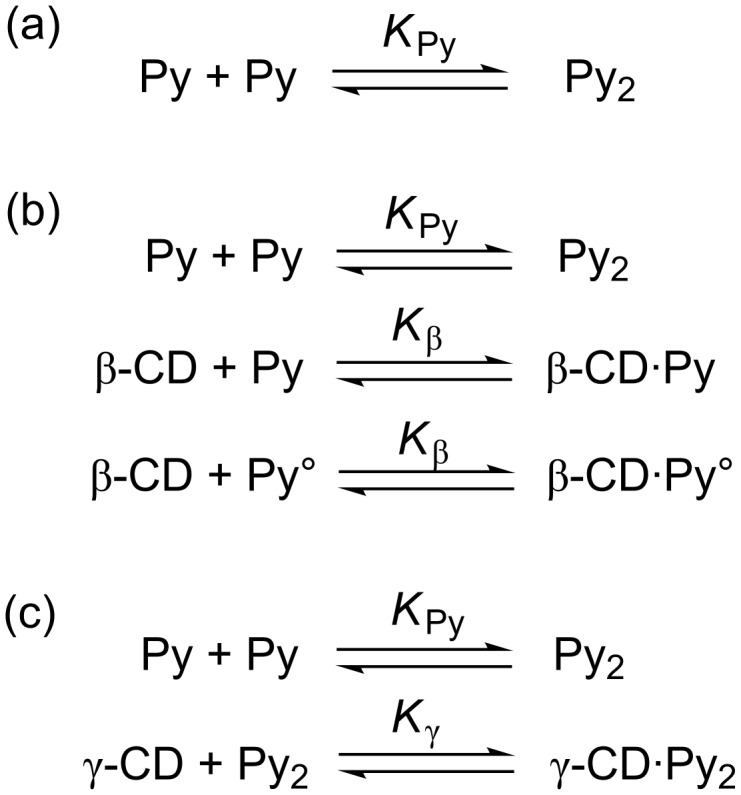
Simplified equilibria of CDs/pAAmPy systems.

Detailed study of the equilibria of the inclusion complex formation of CDs with Py-modified water-soluble polymers, including the formation of the dynamic excimer, requires not only steady-state fluorescence measurements but also time-resolved fluorescence measurements [38–43]. In this study, however, equilibrium constants are roughly estimated by analyzing the steady-state fluorescence data, assuming that dynamic excimer formation is negligible. In the absence of CD, *I*_480_/*I*_376_ for pAAmPy decreases from 0.125 to 0.025 with increasing *x*_DMSO_ from 0 to 1. It should be noted here that the fluorescence of the Py monomer is dominant compared to that of the Py excimer even at *x*_DMSO_ = 0 (i.e., in water), implying that there are a significant fraction of Py residues (Py°) that cannot form Py dimers (Py_2_). Since the steady-state fluorescence measurements were performed under dilute conditions in this study, most of the Py_2_ were formed intramolecularly. Thus, Py residues in pAAmPy carrying a Py residue may not form Py_2_. The fraction of Py° is defined as *f*. Scheme 2a indicates a simplified equilibrium of the formation of Py_2_ from two Py residues. On the basis of the derivation of equations in the Supporting Information File 1, the equilibrium constant for the Py_2_ formation (*K*_Py_) can be calculated as

[1]
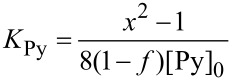


where [Py]_0_ is the total concentration of Py residue and *x* as given in Equation 2.

[2]
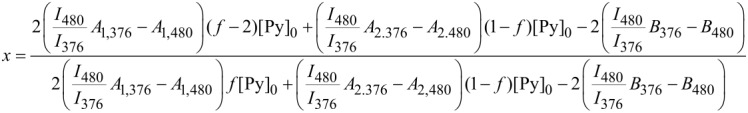


**Figure 4:**
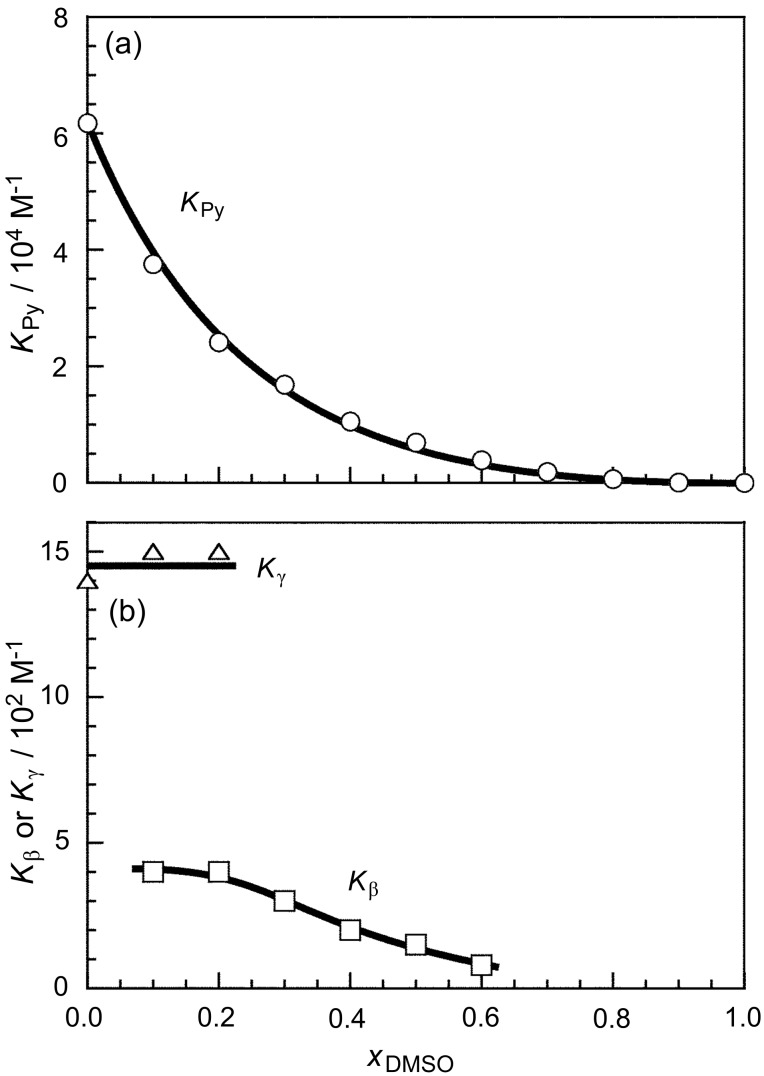
*K*_Py_, *K*_β_, and *K*_γ_ as a function of *x*_DMSO_.

Here, *A*_1,376_, *A*_1,480_, *A*_2,376_, and *A*_2,480_ are constants corresponding to the products of the molar extinction coefficient and the fluorescence quantum yield (subscripts 1 and 2 indicate monomeric and dimeric Py residues, respectively, and subscripts 376 and 480 indicate the wavelengths), and *B*_376_ and *B*_480_ are constants corresponding to the background. If it is assumed that all the Py residues are in the monomer state at *x*_DMSO_ = 1 (i.e., in DMSO), *f* = 0.5, *A*_2,480_/*A*_1,376_ = 0.5, and *B*_376_ = *B*_480_ = 0, *K*_Py_ can be calculated as can be seen in Figure 4a. This figure indicates that *K*_Py_ decreases monotonously from 6.2 × 10^4^ to 0 M^−1^ with increasing *x*_DMSO_ from 0 to 1.

In the β-CD/pAAmPy system, β-CD forms inclusion complexes with both Py and Py° (Scheme 2b). On the basis of the derivation described in the Supporting Information File 1, the concentrations of all species can be calculated by using the equilibrium constant (*K*_β_) for the inclusion complex formation, and *I*_480_/*I*_376_ can be also obtained as given in Equation 3.

[3]
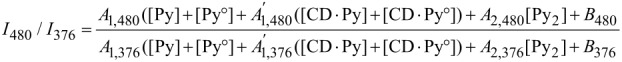


Here [Py], [Py°], [CD], [CD·Py], and [CD·Py°] denote the concentrations of Py, Py°, free CD, and the complexes of CD with Py and with Py°, respectively, and *A'*_1,376_ and *A'*_1,480_ are constants. In this study, *A'*_1,480_/*A'*_1,376_ is fixed at 0.025 (Supporting Information File 1). It is also likely that *A*_2,376_ = 0. When *K*_β_ and *A'*_1,376_/*A*_1,376_ are chosen appropriately, the calculated *I*_480_/*I*_376_ values agree with the experimental data, as can be seen in Figure 3a. The *K*_β_ values were plotted in Figure 4b against *x*_DMSO_. As *x*_DMSO_ increases from 0.1 to 0.6, *K*_β_ decreases from 4 × 10^2^ to 8 × 10^1^ M^−1^. This observation indicates that the formation of inclusion complexes becomes less favorable with increasing *x*_DMSO_.

In the γ-CD/pAAmPy system, γ-CD forms inclusion complexes with Py_2_, in which Py° is not involved. On the basis of the derivation described in the Supporting Information File 1, the concentrations of all species can be calculated by using the equilibrium constant (*K*_γ_) for the inclusion complex formation, and *I*_480_/*I*_376_ can be also obtained as given in Equation 4.

[4]
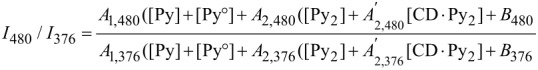


Here [CD·Py_2_] denotes the concentration of the complex of CD with Py_2_, and *A'*_2,480_ is a constant. It is also likely that *A*_2,376_ = *A'*_2,376_ = 0. When *K*_γ_ and *A'*_2,480_/*A*_1,376_ are chosen appropriately, the *I*_480_/*I*_376_ values calculated agree with the experimental data, as can be seen in Figure 3b. The *K*_γ_ values were also plotted in Figure 4b against *x*_DMSO_. This figure indicates that *K*_γ_ is practically constant (ca. 1.5 × 10^3^ M^–1^) independent of *x*_DMSO_ in the region of 0 ≤ *x*_DMSO_ ≤ 0.2.

It should be noted here that the values of *K*_Py_, *K*_β_, and *K*_γ_ were estimated rather qualitatively based on the simplified equilibria and a number of assumptions, but the *K*_β_ and *K*_γ_ values are in good agreement with the values reported for pyrene (4.9 × 10^2^ and 1.1 × 10^3^ M^−1^ for β-CD and γ-CD, respectively) [44].

## Experimental

1-Pyrenemethylamine hydrochloride was purchased from Sigma-Aldrich Co. Ltd. Acryloyl chloride was obtained from Tokyo Chemical Industry Co. Ltd. Triethylamine, acrylamide (AAm), ammonium peroxodisulfate (APS), acetone, methanol, DMSO (spectroscopic grade), NaHCO_3_, and NaOH were purchased from Nacalai Tesque Inc. *N*,*N*-Dimethylformamide (DMF) and dichloromethane (DCM) were purified by utilizing a glass contour solvent dispensing system. Water was purified by a Millipore Milli-Q system. β-CD and γ-CD were purchased from Junsei Chemical Co. Ltd. and recrystallized twice from water before use. *N*-1-Pyrenylmethylacrylamide (APy) was prepared from 1-pyrenemethylamine hydrochloride and acryloyl chloride according to the procedure reported previously [37]. Other reagents were reagent grade and used without further purification.

The polymer (pAAmPy) was prepared by radical copolymerization of AAm and APy using APS as the initiator. A predetermined amount of AAm and APy were dissolved in DMF. After purging with dry argon for 30 min, APS (3 mg, 13 μmol) was added to the monomer solution. The reaction mixture was placed into a cuvette equipped with a stirrer and sealed. The cuvette was warmed with an oil bath thermostated at 60 °C overnight. The reaction mixture was poured into an excess of methanol to give a precipitate. The polymer obtained was recovered by filtration and dried under vacuum. The molecular weight of the polymer was estimated to be 4 × 10^3^ by size exclusion chromatography (SEC), and the Py content was determined to be ca. 1 mol % by ^1^H NMR.

Steady-state fluorescence spectra were obtained on a HITACHI F-2500 spectrophotometer with excitation at 335 nm by using a 1 cm quartz cuvette. The slit widths for both excitation and emission sides were kept at 2.5 nm during measurement. SEC analysis was carried out at 40 °C on a TOSOH CCP & 8020 system equipped with two TOSOH TSKgel α-M columns connected in series, using formamide as the eluent at a flow rate of 0.3 mL min^−1^. TOSOH UV-8020 and TOSOH RI-8021 detectors were used. The molecular weights were calibrated by polystyrene sulfonate sodium-salt samples (American Polymer Standards). ^1^H NMR spectra were measured on a JEOL JNM-ECA500 spectrometer by using a mixed solvent of DMSO-*d*_6_ and D_2_O (1/1, v/v) as a solvent, and chemical shifts were referenced to the solvent value (i.e., 2.49 ppm for DMSO).

## Supporting Information

File 1 Equilibria for the CDs/pAAmPy systems.
