# Supplementary material for: Interaction of cyclodextrins with pyrene-modified polyacrylamide in a mixed solvent of water and dimethyl sulfoxide as studied by steady-state fluorescence
Source: Beilstein J Org Chem. 2012 Aug 16;8:1312–7. doi: 10.3762/bjoc.8.150 (PMC3458755; doi:10.3762/bjoc.8.150)
Supplement: File 1 — Equilibria for the CDs/pAAmPy systems. [file Beilstein_J_Org_Chem-08-1312-s001.pdf]

Supporting Information

for

Interaction of cyclodextrins with pyrene-modified polyacrylamide in a mixed solvent of water and dimethyl sulfoxide as studied by steady-state fluorescence

Akihito Hashidzume[1], Yongtai Zheng[1] and Akira Harada*[1,2]

Address: [1]Department of Macromolecular Science, Graduate School of Science, Osaka University, 1-1 Machikaneyama-cho, Toyonaka, Osaka 560-0043, Japan and [2]Japan Science and Technology Agency (JST), Core Research for Evolutional Science and Technology (CREST), 7 Gobancho, Chiyoda-ku, Tokyo 102-0076, Japan

Email: Akira Harada - harada@chem.sci.osaka-u.ac.jp

* Corresponding author

Equilibria for the CDs/pAAmPy systems

The CD-Free System

Scheme 2a shows a simplified equilibrium in the CD-free system, where Py is the Py residue, which can form Py dimer (Py_2). The equilibrium constant for the Py dimer formation (K_{Py}) is defined as

$$K_{Py} = \frac{[Py_2]}{[Py]^2} \quad \text{(S1)}$$

where [Py] and [Py_2] denote the concentrations of Py monomer and dimer, respectively. Thus,

$$[Py_2] = K_{Py}[Py]^2 \quad \text{(S2)}$$

In this study, a significant fraction of Py residues (Py°) cannot form Py_2, and the mole fraction of Py° is defined as *f*. Based on the conservation of mass,

$$(1 - f)[Py]_0 = [Py] + 2[Py_2] \quad \text{(S3)}$$

where $[Py]_0$ is the total concentration of Py residue. Substituting eq S2 into eq S3, it can be solved for [Py] as

$$[Py] = \frac{-1 + \sqrt{1 + 8K_{Py}(1 - f)[Py]_0}}{4K_{Py}} \quad \text{(S4)}$$

Substituting eq S4 into eq S2,

$$[Py_2] = \frac{1 - \sqrt{1 + 8K_{Py}(1 - f)[Py]_0} + 4K_{Py}(1 - f)[Py]_0}{8K_{Py}} \quad \text{(S5)}$$

When K_{Py}, *f*, and $[Py]_0$ are given, [Py] and [Py_2] can be calculated by using eqs S4 and S5.

It is assumed that the ratio of fluorescence intensities at 480 and 376 nm (I_{480}/I_{376}) can be calculated as

$$I_{480}/I_{376} = \frac{A_{1,480}([Py] + [Py^\circ]) + A_{2,480}[Py_2] + B_{480}}{A_{1,376}([Py] + [Py^\circ]) + A_{2,376}[Py_2] + B_{376}} \quad \text{(S6)}$$

where $A_{1,376}$, $A_{1,480}$, $A_{2,376}$, and $A_{2,480}$ are constants corresponding to the products of the molar extinction coefficient and fluorescence quantum yield (subscripts 1 and 2 indicate monomeric and dimeric Py residues, respectively, and subscripts 376 and 480 indicate the wavelengths), and B_{376} and B_{480} are constants corresponding to the background. Substituting eqs S2 and S4 into eq S6, it can be solved for K_{Py} as

$$K_{Py} = \frac{x^2 - 1}{8(1-f)[Py]_0} \tag{S7}$$

where

$$x = \frac{2\left(\frac{I_{480}}{I_{376}}A_{1,376} - A_{1,480}\right)(f-2)[Py]_0 + \left(\frac{I_{480}}{I_{376}}A_{2,376} - A_{2,480}\right)(1-f)[Py]_0 - 2\left(\frac{I_{480}}{I_{376}}B_{376} - B_{480}\right)}{2\left(\frac{I_{480}}{I_{376}}A_{1,376} - A_{1,480}\right)f[Py]_0 + \left(\frac{I_{480}}{I_{376}}A_{2,376} - A_{2,480}\right)(1-f)[Py]_0 - 2\left(\frac{I_{480}}{I_{376}}B_{376} - B_{480}\right)} \tag{S8}$$

Since I_{480}/I_{337} values are known at different x_{DMSO}, K_{Py} can be calculated at different x_{DMSO} when $A_{1,480}/A_{1,376}$, $A_{2,376}/A_{1,376}$, $A_{2,480}/A_{1,376}$, $B_{376}/A_{1,376}$, $B_{480}/A_{1,376}$, and f are given. For simplicity, it is assumed here that $A_{2,376} = 0$ and $B_{376} = B_{480} = 0$. Assuming that all the Py residues are in the monomer state at $x_{DMSO} = 1$, $A_{1,480}/A_{1,376} = 0.025$ (= I_{480}/I_{376} at $x_{DMSO} = 1$). In this study, both $A_{2,480}/A_{1,376}$ and f are fixed at 0.5.

The β-CD/pAAmPy System

Scheme 2b shows simplified equilibria for the β-CD/pAAmPy system, where CD•Py and CD•Py° are inclusion complexes. The equilibrium constants (K_{Py} and K_β) are expressed as

$$K_{Py} = \frac{[Py_2]}{[Py]^2} \tag{S9}$$

$$K_\beta = \frac{[CD \bullet Py]}{[CD][Py]} = \frac{[CD \bullet Py^\circ]}{[CD][Py^\circ]} \tag{S10}$$

where [Py], [Py°], [CD], [CD•Py], and [CD•Py°] denote the concentrations of Py, Py°, free CD, the complexes of CD with Py and with Py°, respectively. From eqs S9 and S10,

$$[Py_2] = K_{Py}[Py]^2 \tag{S11}$$

$$[CD\bullet Py] = K_{\beta}[CD][Py] \tag{S12}$$

$$[CD\bullet Py^{\circ}] = K_{\beta}[CD][Py^{\circ}] \tag{S13}$$

Based on the conservation of mass,

$$(1 - f)[Py]_0 = [Py] + 2[Py_2] + [CD\bullet Py] \tag{S14}$$

$$f[Py]_0 = [Py^{\circ}] + [CD\bullet Py^{\circ}] \tag{S15}$$

$$[CD]_0 = [CD] + [CD\bullet Py] \tag{S16}$$

where $[CD]_0$ is the total concentration of CD. Substituting eqs S11 and S12 into eq S14, it can be solved for [Py] as

$$[Py] = \frac{-(1+K_{\beta}[CD]) + \sqrt{(1+K_{\beta}[CD])^2 + 8K_{Py}(1-f)[Py]_0}}{4K_{Py}} \tag{S17}$$

Substituting eq S13 into eq S15, it can be solved for [Py°] as

$$[Py^{\circ}] = \frac{f[Py]_0}{1+K_{\beta}[CD]} \tag{S18}$$

When K_{Py}, K_{β}, [CD], and $[Py]_0$ are given, $[Py_2]$, [CD•Py], and [CD•Py°] can be calculated by substituting eqs S17 and S18 into eqs S11 – S13.

I_{480}/I_{376} can be calculated as

$$I_{480}/I_{376} = \frac{A_{1,480}([Py]+[Py^{\circ}]) + A'_{1,480}([CD\bullet Py]+[CD\bullet Py^{\circ}]) + A_{2,480}[Py_2] + B_{480}}{A_{1,376}([Py]+[Py^{\circ}]) + A'_{1,376}([CD\bullet Py]+[CD\bullet Py^{\circ}]) + A_{2,376}[Py_2] + B_{376}} \tag{S19}$$

where $A'_{1,376}$ and $A'_{1,480}$ are also constants. In this study, $A'_{1,480}/A'_{1,376}$ is also fixed at 0.025. It is also likely that $A_{2,376} = 0$. The free CD concentration ([CD]) is necessary for these calculations, but only $[CD]_0$ is known in experiments. Thus, upon the fitting procedure, $[CD]_0$ values calculated numerically with eq S16 were used.

The γ-CD/pAAmPy System

Scheme 2c shows simplified equilibria for the γ-CD/pAAmPy system. The equilibrium constants (K_{Py} and K_{γ}) are expressed as

$$K_{\mathrm{Py}} = \frac{[\mathrm{Py}_2]}{[\mathrm{Py}]^2} \tag{S20}$$

$$K_{\gamma} = \frac{[\mathrm{CD} \bullet \mathrm{Py}_2]}{[\mathrm{CD}][\mathrm{Py}_2]} \tag{S21}$$

where [CD•Py_2] denotes the concentration of the complex of CD with Py_2. From eqs S20 and S21,

$$[\mathrm{Py}_2] = K_{\mathrm{Py}}[\mathrm{Py}]^2 \tag{S22}$$

$$[\mathrm{CD}\bullet\mathrm{Py}_2] = K_{\gamma}[\mathrm{CD}][\mathrm{Py}_2] \tag{S23}$$

Based on the conservation of mass,

$$(1 - f)[\mathrm{Py}]_0 = [\mathrm{Py}] + 2[\mathrm{Py}_2] + 2[\mathrm{CD}\bullet\mathrm{Py}_2] \tag{S24}$$

$$[\mathrm{CD}]_0 = [\mathrm{CD}] + [\mathrm{CD}\bullet\mathrm{Py}_2] \tag{S25}$$

Substituting eqs S22 and S23 into eq S24, it can be solved for [Py] as

$$[\mathrm{Py}] = \frac{-1 + \sqrt{1 + 4(K_{\mathrm{Py}} + K_{\mathrm{Py}}K_{\gamma}[\mathrm{CD}])(1-f)[\mathrm{Py}]_0}}{2(K_{\mathrm{Py}} + K_{\mathrm{Py}}K_{\gamma}[\mathrm{CD}])} \tag{S26}$$

When K_{Py}, K_{γ}, [CD], and $[\mathrm{Py}]_0$ are given, [Py_2] and [CD•Py_2] can be calculated by substituting eq S26 into eqs S22 and S23.

I_{480}/I_{376} can be calculated as

$$I_{480}/I_{376} = \frac{A_{1,480}([\mathrm{Py}] + [\mathrm{Py}^{\circ}]) + A_{2,480}[\mathrm{Py}_2] + A'_{2,480}[\mathrm{CD}\bullet\mathrm{Py}_2] + B_{480}}{A_{1,376}([\mathrm{Py}] + [\mathrm{Py}^{\circ}]) + A_{2,376}[\mathrm{Py}_2] + A'_{2,376}[\mathrm{CD}\bullet\mathrm{Py}_2] + B_{376}} \tag{S27}$$

where $A'_{2,480}$ is also a constant. It is also likely that $A_{2,376}$ = $A'_{2,376}$ = 0. The free CD concentration ([CD]) is necessary for these calculations, but only $[\mathrm{CD}]_0$ is known in experiments. Thus, upon the fitting procedure, $[\mathrm{CD}]_0$ values calculated numerically with eq S25 were used.
